# Upregulation of Far Upstream Element-Binding Protein 1 (FUBP1) Promotes Tumor Proliferation and Tumorigenesis of Clear Cell Renal Cell Carcinoma

**DOI:** 10.1371/journal.pone.0169852

**Published:** 2017-01-11

**Authors:** Junyao Duan, Xu Bao, Xin Ma, Yu Zhang, Dong Ni, Hanfeng Wang, Fan Zhang, Qingshan Du, Yang Fan, Jianwen Chen, Shengpan Wu, Xintao Li, Yu Gao, Xu Zhang

**Affiliations:** 1 School of Medicine, Nankai University, Tianjin, China; 2 Department of Urology, State Key Laboratory of Kidney Diseases, Chinese People’s Liberation Army General Hospital, PLA Medical School, Beijing, China; Sapporo Ika Daigaku, JAPAN

## Abstract

**Objective:**

The far upstream element (FUSE)-binding protein 1 (FUBP1) is a transactivator of human *c-myc* proto-oncogene transcription, with important roles in carcinogenesis. However, the expression pattern and potential biological function of FUBP1 in clear cell renal cell carcinoma (ccRCC) is yet to be established.

**Methods:**

FUBP1 expression was detected in ccRCC tissues and cell lines by real-time RT-PCR, Western blot analysis, and immunohistochemistry. The correlations of FUBP1 mRNA expression levels with clinicopathological factors were evaluated. The biological function of FUBP1 during tumor cell proliferation was studied by MTS, colony formation, and soft-agar colony formation. The effects of FUBP1 on cell cycle distribution and apoptosis were analyzed by flow cytometry. Western blot analysis was used to identify the potential mechanism of FUBP1 regulating cell cycle and apoptosis.

**Results:**

The levels of FUBP1 mRNA and protein expression were upregulated in human ccRCC tissues compared with adjacent noncancerous tissues. High levels of FUBP1 mRNA expression were associated with higher tumor stage and tumor size. FUBP1 knockdown inhibited cell proliferation and induced cell cycle arrest and apoptosis. Meanwhile, the expression levels of c-myc and p21 mRNA were correlated with that of FUBP1 mRNA.

**Conclusions:**

FUBP1 acts as a potential oncogene in ccRCC and may be considered as a novel biomarker or an attractive treatment target of ccRCC.

## Introduction

Renal cell carcinoma (RCC) has become one of the most lethal urological malignant tumors, which accounted for 62,700 new cases and 14,240 deaths in the United States in 2016. RCC morbidity and mortality rate has continuously increased from 2003 to 2012 [1]. Although more RCC cases are detected at an earlier stage, 20%–30% of patients present metastasis. The recurrence rates are 20%–40% after curative surgical resection. Median survival of advanced-stage RCC patients is only 18–24 months. With the present target-specific drugs, the percentage of patients achieving an objective response is between 20% and 40%, but complete response is only seen in 1%–3% of patients, and patients with high-risk RCC receive no benefit from adjuvant sunitinib or sorafenib treatment [2–4]. Even with strict adherence to the current 2014 NCCN and AUA renal cell carcinoma surveillance guidelines, approximately one-third of all recurrences are missed [5]. RCC consists of numerous histological, molecular, and clinically distinct malignant tumors, which originate from the nephron. Clear cell renal cell carcinoma (ccRCC) originates from the proximal tubule and represents the most common and aggressive malignant subtype of RCC [6–8]. Therefore, the molecular mechanisms underlying ccRCC initiation and progression should be understood to identify novel therapeutic targets or biomarkers.

The far upstream element-binding protein 1 (FUBP1) contributes to the c-myc transcriptional regulation was first identified from undifferentiated HL60 cells in 1994, which was mapped to chromosome 1p31.1 [9, 10]. FUBP1 is composed of three functional domains: a repression amino-terminal domain, a DNA-binding central domain, and a transactivation carboxyl-terminal domain. The DNA-binding central domain consists of four KH motifs which could unwind the helix of far upstream element (FUSE) and specifically bind to the noncoding strand. The tyrosine-rich carboxyl-terminal domain could physically interact with the general transcription factor IIH (TFIIH) and promote transcription. The amino-terminal domain could repress the activity of the carboxyl-terminal domain [11–14]. FUBP1 is broadly expressed in a wide variety of tissues and cells with a distinct spatial temporal profile where it influences a number of cellular processes, including differentiation, cell proliferation, apoptosis, or cell death through the effects of transcription, mRNA stability, and translation [12, 15–18]. Increasing evidence suggests that FUBP1 is overexpressed in a variety of malignancies, including prostate cancer, bladder cancer, liver cancer, colon cancer, breast cancer, non-small-cell lung cancer (NSCLC), glioma, and gastric cancer [19–30]. FUBP1 is also involved in tumor initiation and progression by affecting several key cellular activities of tumor cells, such as apoptosis, proliferation and migration [11]. For example, FUBP1 is strongly overexpressed in human hepatocellular carcinoma and is necessary for resistance to apoptotic stimuli and cell proliferation by direct or indirect repression of cell cycle inhibitors and proapoptotic target genes [24]. In U2OS cells, FUBP1 knockdown could arrest cellular proliferation by extinguishing c-myc expression [31].

Despite several published reports that addressed the role of FUBP1 in promoting cancer development, little is known about its involvement in ccRCC. In the present study, we examined the expression of FUBP1 in ccRCC tissues, as well as elucidated its role in regulating ccRCC proliferation and carcinogenesis. We further monitored the possible molecular mechanism with respect to c-myc and p21 in the context of FUBP1-mediated ccRCC.

## Materials and Methods

### Ethics statement

This study was approved by the Ethics Committee of Chinese People’s Liberation Army (PLA) General Hospital. All patients signed an informed written consent for participation in the study. All the research was conducted based on the Declaration of Helsinki and International Conference on Harmonisation-Good Clinical Practice.

### Patients and samples

A total of 56 pairs of ccRCC tissue samples and matched adjacent noncancerous tissue samples were obtained from patients who underwent nephrectomy at the Urology Department of Chinese PLA General Hospital (Beijing, China) between 2013 and 2014. All samples were preserved by flash freezing in liquid nitrogen after resection and stored at −80°C. The samples were histologically diagnosed by two senior pathologists. TNM staging of ccRCC samples were performed according to the 7th edition of the AJCC Cancer Staging Manual, and the Fuhrman nuclear grading system was used to determined nuclear grades. Patient characteristics are provided in Table 1.

**Table 1:** Correlation between FUBP1 expression and clinicopathological characteristics of ccRCC.

Characteristics	n	FUBP1 expression		*P* value
		Low	High	
**Total cases**	56	32	24	
**Gender**				
Male	34	16	18	0.0580^a^
Female	22	16	6	
**Age (years)**				
<60	31	21	10	0.0743^a^
≥60	25	11	14	
**BMI**				
<25	18	11	7	0.6796^a^
≥25	38	21	17	
**Tumor size (cm)**				
≤7	41	27	14	0.0294^a^
>7	15	5	10	
**TNM stage**				
I/II	45	30	15	0.0101^b^
III	11	2	9	
**Fuhrman**				
I/II	46	27	19	0.8799^b^
III	10	5	5	

^a^ Chi-square test

^b^ Chi-square test with Yates’ correction

### Cell lines and culture

The human renal proximal tubule epithelial cell line HKC and the human ccRCC cell lines, including 786-O and caki-1, were purchased from the National Platform of Experimental Cell Resources for Sci-Tech (Beijing, China). HKC, 786-O, and caki-1 were cultured in RPMI 1640, RPMI 1640, and high-glucose DMEM (Hyclone, USA), respectively, supplemented with 10% fetal bovine serum (TransGen Biotech, Beijing, China), 100 U/mL penicillin, and 100 μg/mL streptomycin (Solarbio, Beijing, China). All the cells were maintained at 37°C in a humidified incubator with 5% CO_2_.

### Quantitative reverse-transcription polymerase chain reaction (qRT-PCR)

Total RNA was isolated from tissue samples and cell lines with the TRIzol Reagent (ComWin Biotech, Beijing, China) following the manufacturer’s protocol. cDNA was synthesized with the TransScript First-Strand cDNA Synthesis SuperMix (TransGen Biotech, Beijing, China) according to the manufacturer’s instructions. qRT-PCR was conducted on the Applied Biosystems 7500 Detection System (Applied Biosystems, Foster City, CA) with the TransStart Green qPCR SuperMix (TransGen Biotech, Beijing, China) and the following primers: (1) FUBP1 forward 5´-TAGCAAGGCAGGATTAGTC-3´ and reverse 5´-CACGAATTAACTCTAACACCAT-3´; (2) PPIA forward 5´-ATGGTCAACCCCACCGTGT-3´ and reverse 5´-TCTGCTGTCTTTGGGACCTTGTC-3´. The reaction conditions were as follows: 94°C for 30 s followed by 40 cycles at 94°C for 5 s and 60°C for 34 s. The relative expression was quantified by the 2^-ΔΔCt^ method [32] and was normalized to the expression levels of housekeeping gen PPIA. All experiments were performed in triplicates.

### Western blot analysis

Tissues and cells were lysed in RIPA buffer with protease inhibitors (Solarbio, Beijing, China). Lysates were incubated on ice for 30 min then centrifuged at 12,000 rpm for 5 min. Protein concentrations were quantitated by BCA Protein Detection reagent (Applygen Technologies, Beijing, China). Approximately 50 mg of the protein lysates were resuspended in 5× SDS-PAGE sample/loading buffer (Applygen Technologies, Beijing, China) and boiled at 95°C for 10 min. Lysates were loaded onto 5%–10% SDS polyacrylamide gels (Solarbio, Beijing, China), separated by electrophoresis, and electrophoretically transferred onto polyvinylidene difluoride membranes (Millipore, Billerica, MA). The membranes were blocked in Tris-buffered saline-Tween (TBST) with 5% skim milk (BD Bioscience, USA) at 37°C for 2 h. Thereafter, the membranes were incubated overnight at 4°C with the primary antibodies against FUBP1 (1:2000 membrane to primary antibody ratio) (Abcam, Cambridge, MA, USA) or β-actin (1:3000 membrane to β-actin ratio) (ZSGB-BIO, Beijing, China). The membranes were then washed three times for 10 min each time with TBST and incubated with either anti-rabbit or anti-mouse horseradish peroxidase-conjugated secondary antibodies (1:5000 membrane to secondary antibody ratio) (ZSGB-BIO, Beijing, China) at 37°C for 1 h. The membranes were washed thrice with TBST and then developed with an enhanced chemiluminescence detection system (Thermo Fisher Scientific, USA). β-actin was used as a loading control.

### Immunohistochemistry

A total of 49 pairs of 4 μm-thick tissue sections of ccRCC patients sliced from paraffin-embedded blocks were deparaffinized twice with xylene and rehydrated in graded alcohol. Antigen retrieval was achieved by incubating the slides in 10 mM sodium citrate buffer (pH 6.0) at 95°C for 10 min, followed by cooling at room temperature and washing in tap water. Endogenous peroxidase activity was blocked by incubating the samples in 3% hydrogen peroxide solution at 37°C for 20 min. The slides were immersed in phosphate-buffered saline (PBS) thrice for 5 min each time within normal goat serum to the block nonspecific reaction. The slides were then incubated with the primary antibody FUBP1 (1:1000 slide to FUBP ratio; Abcam, Cambridge, MA, USA) overnight at 4°C in a humidified chamber. After immersion within PBS, the slides were processed with the DAB Detection Kit (ZSGB-BIO, Beijing, China) according to the manufacturer’s instructions. Two pathologists blinded to the clinical data independently scored the tissue staining. The German semiquantitative scoring system was used. A value was assigned to each specimen according to the intensity of nucleic staining (no staining = 0, weak staining = 1, moderate staining = 2, and strong staining = 3) and the percentage of staining (0% = 0, 1%–24% = 1, 25%–49% = 2, 50%–74% = 3, and 75%–100% = 4). The final staining index was determined by multiplying the intensity value with the percentage value, which ranged from 0 to 12. We defined 0 index as negative and 1–12 as positive.

### Oligonucleotides and transfection

Three siRNAs (siRNA1: 5´-CCGAAAGGAUAGCACAAAUTT-3´, siRNA2: 5´-GCAGGUGCACCAACUACAATT-3´, and siRNA3: 5´-CCCUGCACCUUAUAAUCCUTT-3´) targeting FUBP1 mRNA also were designed and synthesized by GenePharma (Shanghai, China). When the confluence of cells reached to 70%–90%, siRNAs were transfected at a final concentration of 100 nM with Lipofectamine 2000 (Invitrogen) according to the manufacturer’s protocol. Knockdown levels of each siRNA were determined by Western blot analysis.

### Cell proliferation assay

The assay was performed with the CellTiter 96^®^ AQueous One Solution Cell Proliferation Assay Kit (Promega, USA) according to the manufacturer’s instruction. After transfection, 2×10^3^ cells were seeded in 96-well plates and immediately cultured for 24, 48, 72, and 96 h. A total of 20 μL of the reagent was added into each well and incubated at 37°C for 2 h. The absorbance at 490 nm was measured on a microplate reader (BioTek Instruments, USA).

### Colony formation assay

The assay was performed as previously described in detail [33], with some modifications. The 786-O and caki-1 transfected with siRNAs for 48 h were immediately plated in 6-well plates at 400–1000 cells/well then cultured for 2 weeks. The cells were fixed and stained for 30 min in 35% methanol solution with 1% crystal violet. The area percentage covered by the colonies were quantitated with the ImageJ software with a plugin “ColonyArea” [34].

### Soft agar colony formation assay

A total of 1 mL 0.5% agar in complete culture medium was added into each well of a 6-well plate. A total of 1 mL 0.3% agar solution (1000 cells/mL) was layered over the bottom agar. Plates were incubated at 37°C and were fed every 3 to 4 days with 500 μL of complete medium. The colonies were counted after 3–4 weeks.

### Cell cycle analysis

The assay was performed with the DNA Content Quantitation Assay Kit (Solarbio, Beijing, China) according to the manufacturer’s instruction. The 786-O and caki-1 cells were immediately cultured in 6-well plates for 48 h after transfection with siRNAs. The cells were then washed with ice-cold PBS, suspended in 70% ethanol, and incubated overnight at −4°C. After fixation, the cells were washed twice with ice-cold PBS and maintained at 37°C for 30 min with 100 μL RNase. The cells were stained with 400 μL PI per tube for 30 min in the dark at room temperature. Subsequently, the cell cycle profile was analyzed with a BD FACSCalibur flow cytometer (Becton Dickinson, Franklin Lakes, NJ).

### Apoptosis analysis

At 48 h after transfection, 786-O and caki-1 cells were harvested, washed, stained with annexin V-FITC/PI (Solarbio, Beijing, China), and its concentration was determined by flow cytometry.

### Statistical analysis

Statistical analysis was performed with the GraphPad Prism software, version 6.0. The Chi-square test was used to analyze the correlation between clinicopathological characteristics and levels of FUBP1 mRNA expression. The correlation between t levels of c-myc or p21 mRNA expression and that of FUBP1 mRNA was analyzed by Pearson test. Statistical differences between two groups were analyzed by a paired, two-tailed Student’s *t*-test. Data were represented as mean ± standard deviation (SD). A *P* < 0.05 was considered statistically significant.

## Results

### FUBP1 is upregulated in ccRCC tissues and cell lines and correlates with the clinicopathological characteristics

**Fig 1:**
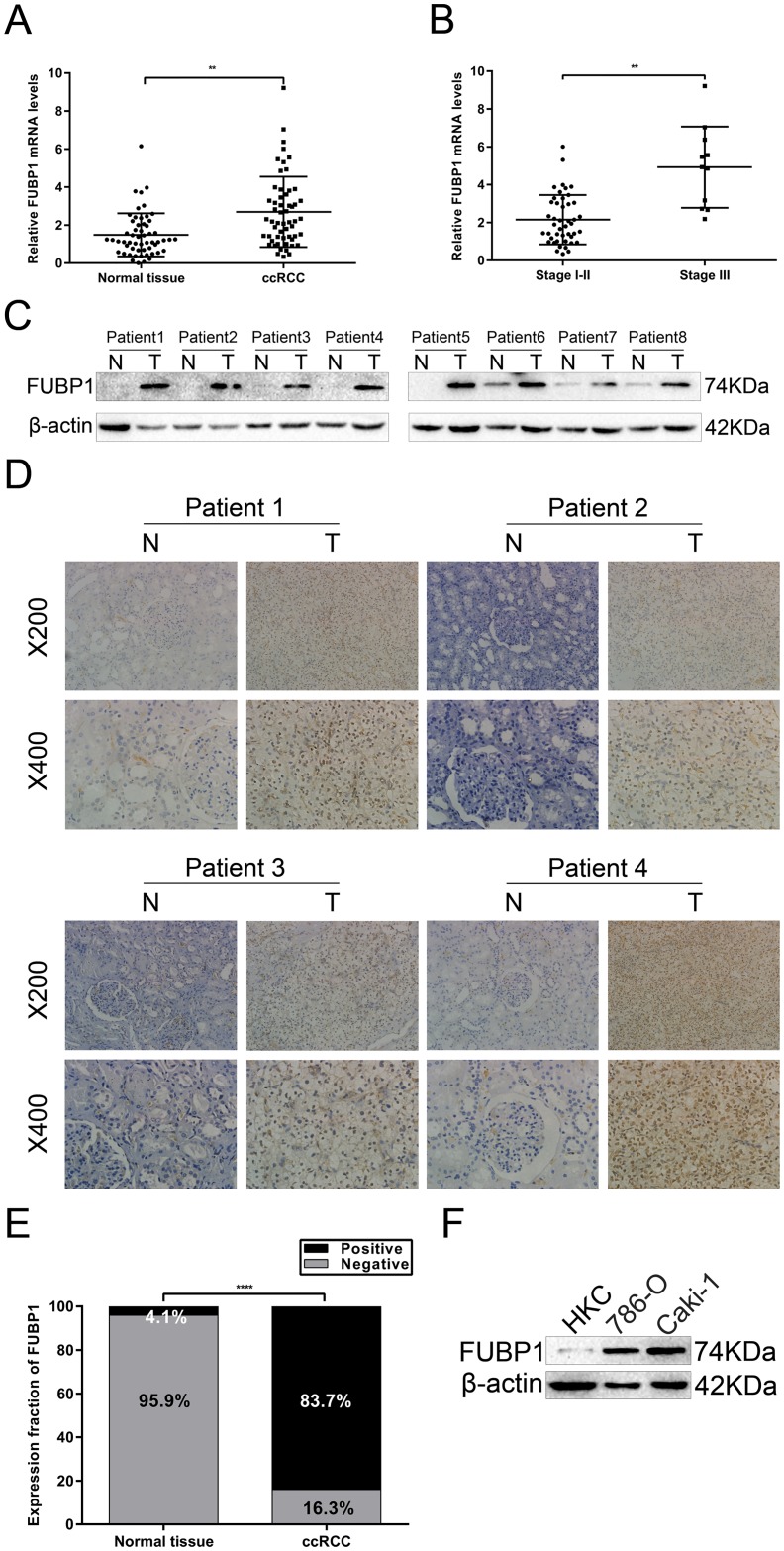
FUBP1 is upregulated in ccRCC tissue and cell lines. (A) FUBP1 mRNA levels in ccRCC and matched normal renal tissues were analyzed by qRT-PCR. The expression levels of FUBP1 mRNA were normalized to that of PPIA, the internal control. (B) Correlation between FUBP1 mRNA levels and TNM stage of ccRCC. (C) Western blot analysis of FUBP1 protein expression in ccRCC (T) and paired normal tissues (N). (D) Representative immunohistochemistry of ccRCC and corresponding adjacent normal renal tissues. Magnification: 200× or 400×. FUBP1 was localized almost exclusively to the nuclei of cells in ccRCC and matched normal renal tissues. (E) FUBP1 protein levels in ccRCC and adjacent normal renal tissues were determined by IHC assays. (F) Western blot analysis of FUPB1 protein expression in HKC, 786-O, and caki-1 cells indicate that FUBP1 protein levels in ccRCC cell lines are significantly higher than that in the HKC cell line. ****P < 0.0001.

To investigate the levels of FUBP1 mRNA and protein expression in ccRCC tissues and cell lines, we performed qRT-PCR, Western blot analysis, and IHC. qRT-PCR analysis of 56 pairs of tissues showed that the levels of FUBP1 mRNA expression were markedly higher in ccRCC tissues than in the matched adjacent normal renal tissues (Fig 1A) and increased along with the progression of TNM stage (Fig 1B). Similarly, Western blot analysis of 8 pairs of tissues revealed that the levels of FUBP1 protein expression were significantly increased in ccRCC tissues compared with the corresponding adjacent normal renal tissues (Fig 1C). In the IHC assay, we also found that the levels of FUBP1 protein expression were significantly upregulated in ccRCC tissues as compared with the matched normal renal tissues from the same patient, FUBP1 was almost exclusively localized in the nucleus of ccRCC cells (Fig 1D and 1E). Furthermore, ccRCC cell lines expressed significantly higher levels of FUBP1 protein compared with that in the renal proximal tubule epithelial cells HKC (Fig 1F). Thus, FUBP1 expression at the protein and mRNA levels was increased in ccRCC tissues and cell lines.

Next, to evaluate the clinical relevance of FUBP1 mRNA expression, we analyzed the association between FUBP1 mRNA expression and the clinicopathological features of ccRCC patients. There are 34 male and 22 female patients. The average (± standard deviation) age at diagnosis was 56.6 ± 12.0 years. The average (± standard deviation) body mass index (BMI) was 26.6 ± 4.3. The median (range) pathological tumor dimension was 4.5 (2.0–12.0) cm. TNM stage was as follows: 45 stage I/II, and 11 stage II/III. According to Fuhrman, 46 cases were grade I/II, and 10 cases grade III. Based on the 2^-ΔΔCt^ values of FUBP1 mRNA with a cut-off value of their mean 2^-ΔΔCt^ value, the 56 ccRCC patients were divided into low-expression and high-expression groups. As shown in Table 1, FUBP1 mRNA expression was markedly associated with tumor size (*P* = 0.0294) and TNM stage (*P* = 0.0101). However, its expression was not associated with patient sex, age, BMI, or pathological grade. Taken together, these results suggested that the high expression of FUBP1 contributed to ccRCC cell proliferation.

### FUBP1 is involved in proliferation of ccRCC cells

**Fig 2:**
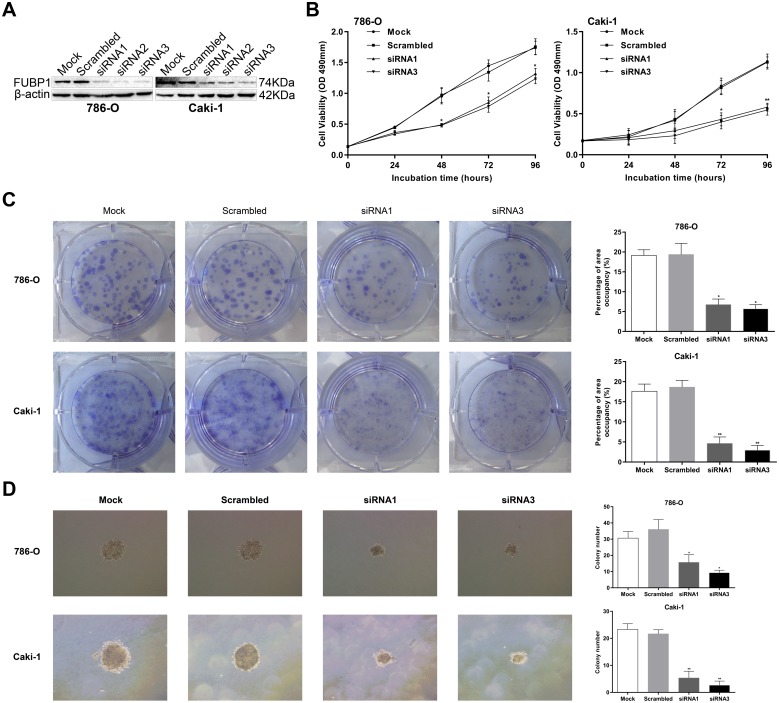
FUBP1 depletion decreases cell proliferation of 786-O and caki-1. (A) FUBP1 was knocked down in 786-O and caki-1 cells, and FUBP1 protein levels were monitored by Western blot analysis with the anti-FUPB1 antibody. β-actin was used as the loading control. (B) Assessment of 786-O and caki-1 cell proliferation by MTS assays. (C) Colony formation assays were performed to determine the proliferation of 786-O siRNA1/3, caki-1 siRNA1/3, and the corresponding control cells (786-O-Mock/Scrambled and caki-1-Mock/Scrambled). The area percentage occupied by 786-O-siRNA1/3 and caki-1-siRNA1/3 cells was markedly less than those of 786-O-Mock/Scrambled and caki-1-Mock/Scrambled. (D) Representative images of soft agar colony assay in cells transfected with indicated siRNAs. Original magnification: 100×. Colony count statistics showed a significant reduction in siRNA1/3 786-O and caki-1 cells. Values are expressed as the mean ± SD of three independent experiments, *P < 0.05, **P < 0.01.

To determine the biological role of FUBP1 in the proliferation of human ccRCC cells, we used chemically synthesized siRNAs, which were designed to target distinct sites of FUBP1 mRNA for knocking down endogenous FUBP1 in ccRCC cell lines of 786-O and caki-1. To exclude the off-target effects, we designed three different siRNAs, Western blot analysis confirmed that all were considered efficient for FUBP1 knockdown (Fig 2A). siRNA1 and siRNA3 were used for the subsequent experiments. Compared with the control cells, 786-O and caki-1 cells transiently transfected with siRNA1 and siRNA3 had markedly inhibited proliferation of ccRCC cells, as assessed by MTS assays (Fig 2B). Furthermore, we examined the capability of cells to form tumor colonies in vitro by a colony formation assay. As expected, the area percentage occupied by siRNA-treated 786-O and caki-1 cells was less than that of the control cells. Colony count statistics demonstrated that the capability of the siRNA-treated 786-O and caki-1 cells to form tumor colonies remarkably decreased (Fig 2C). In addition, we also observed a remarkable decrease in colonies in siRNA-treated 786-O and caki-1 cells at soft agar colony formation assays (Fig 2D). Thus, FUBP1 promoted proliferation of ccRCC cells.

### FUBP1 knockdown arrests 786-O and caki-1 cell proliferations at the G1-S phase transition

**Fig 3:**
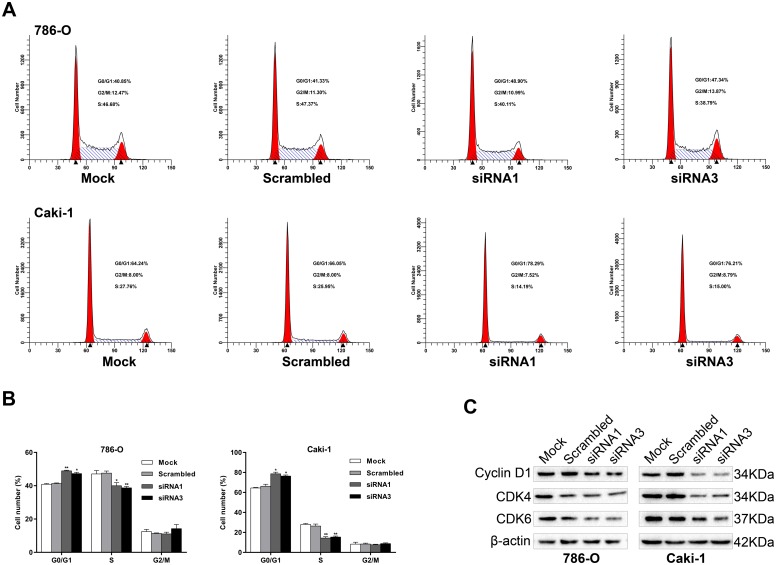
Effects of FUBP1 on cell cycle arrest and levels of cell cycle regulator expression in 786-O and caki-1 cells. (A and B) Representative data from flow cytometry analysis of the cell cycle distribution in 786-O-siRNA1/3, caki-1-siRNA1/3, and the corresponding control cells (786-O-Mock/Scrambled and caki-1-Mock/Scrambled). The percentage of cells in the G0/G1, S, and G2/M phases was quantified and plotted. (C) Western blot analysis of cyclin D1, CDK4, and CDK6. β-actin was used as the loading control. Values are expressed as the mean ± SD of at least three independent experiments. *P < 0.05, **P < 0.01.

To understand the mechanism by which FUBP1 enhanced ccRCC cell proliferation, we performed flow cytometry to measure the cell cycle distribution of siRNA-treated 786-O and caki-1 cells. We found that FUBP1 knockdown increased the percentage of cells in the G0/G1 peak and decreased the percentage of cells in the S peak as compared with those in the matched controls (Fig 3A and 3B). Consistent with the flow cytometry data, the expression of the G1–S phase checkpoint proteins, including cyclin D1, CDK4, and CDK6, was markedly downregulated in 786-O and caki-1 cells that were transiently transfected with siRNA1 and siRNA3 as compared with the control cells (Fig 3C). Collectively, these results indicated that FUBP1 promoted cell proliferation by facilitating cell cycle progression.

### FUBP1 inhibited cell apoptosis of 786-O and caki-1

**Fig 4:**
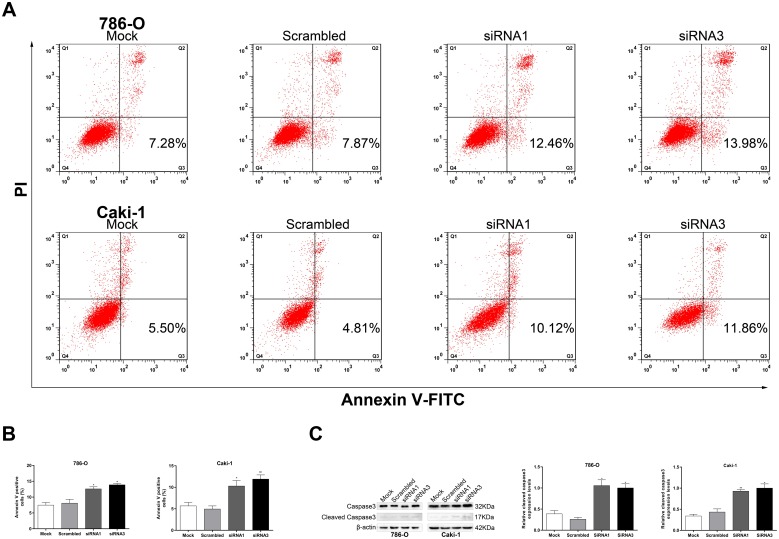
Effects of FUBP1 on cell apoptosis and expression levels of apoptosis-related proteins in 786-O and caki-1 cells. (A and B) 786-O and caki-1 cells transfected with FUBP1 siRNAs were stained with a combination of annexin V and PI and analyzed by FACS. Cells positive for annexin V staining were counted as apoptotic cells, and the percentage of apoptotic cells is shown. (C) Western blot analysis showing the levels of caspase-3 and cleaved caspase-3 expression after FUBP1 knockdown in 786-O and caki-1 cells. Values are expressed as the mean ± SD of at least three independent experiments. *P < 0.05, **P < 0.01.

To investigate whether FUBP1 affects apoptosis of ccRCC cells, we detected cell apoptosis by flow cytometry in 786-O and caki-1 cells stained with annexin V-FITC and PI. The data showed that FUBP1 knockdown of 786-O and caki-1 cells had a remarkably higher percentage of annexin V—FITC-positive cells than that of the control cells (Fig 4A and 4B). Consistent with the flow cytometry data, the expression levels of well-defined apoptosis protein markers, such as cleaved caspase 3, increased in the 786-O and caki-1 cells with the knockdown of FUBP1 expression (Fig 4C). Taken together, FUBP1 inhibited apoptosis in ccRCC cells.

### c-myc and p21 mRNA expression levels are correlated with FUBP1 in ccRCC tissues

**Fig 5:**
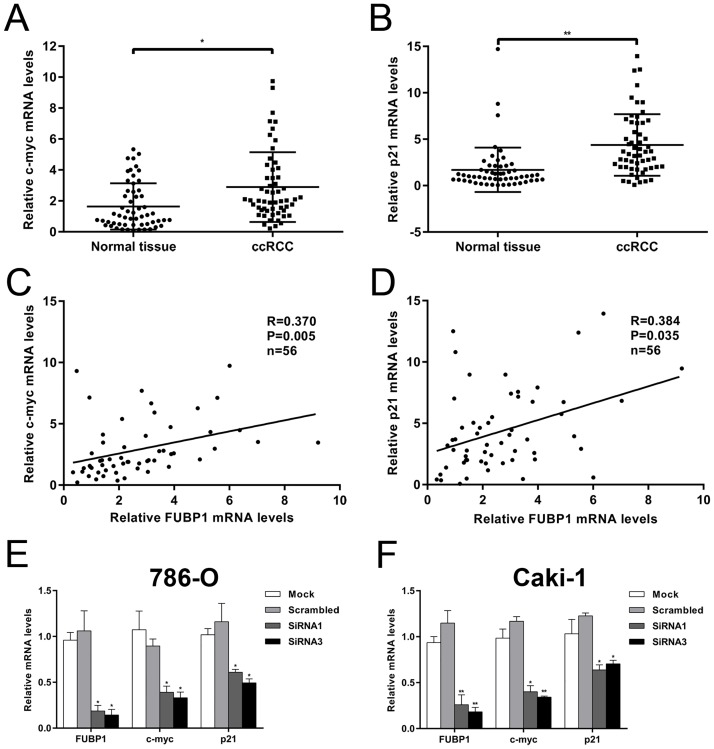
Levels of c-myc and p21 expression are positively correlated with that of FUBP1 in ccRCC tissues. The levels of c-myc mRNA (A) and p21 mRNA (B) expression in 56 human ccRCC tissues and the corresponding adjacent normal tissues were analyzed by qRT-PCR. The correlation between c-myc mRNA (C) or p21 mRNA (D) and the FUBP1 mRNA expression in tissues was detected by qRT-PCR. The mRNA levels of FUBP1, c-myc and p21 were showed after FUBP1 knockdown in 786-O (E) and caki-1 (F) cells. Values are expressed as the mean ± SD of at least three independent experiments. *P < 0.05, ** *P* < 0.01.

To explore the molecular mechanism by which FUBP1 is associated with ccRCC progression, the correlations between FUBP1 mRNA and the c-myc and p21 mRNA were studied by qRT-PCR. We observed that c-myc and p21 mRNA expression were markedly higher in ccRCC tissues than in matched adjacent nontumor tissues (Fig 5A and 5B). A Pearson test revealed that c-myc and p21 mRNA expression was positively correlated with that of FUBP1 mRNA in ccRCC tissues (Fig 5C and 5D). Moreover, after knockdown FUBP1 in 786-O and caki-1 cells, the mRNA levels of c-myc and p21 were significantly decreased (Fig 5E and 5F).

## Discussion

This study revealed that FUBP1 was upregulated in ccRCC patients. In addition, FUBP1 upregulation was significantly correlated with the clinicopathological features of ccRCC, such as tumor size and stage. We also observed that FUBP1 promoted cell proliferation by activating cell cycle progression and inhibiting apoptosis, which is accompanied by changes in the expression of cell cycle and apoptosis regulators. Mechanistically, FUBP1 might increase ccRCC cell growth by activating c-myc or/and p21. These findings indicated that FUBP1 might play an important role in the initiation and development of ccRCC.

Previous studies have reported that FUBP1 is associated with the clinicopathological features and prognosis. In human gliomas, FUBP1 levels were positively correlated with the pathologic stage, the high levels of FUBP1 expression were associated with shorter survival [30]. Malz et al. reported that HCC patients with high levels of FUBP1-1/2 expression had significantly poorer cumulative survival [21]. Zhang et al. reported that if stratified by FUBP1 expression, the overall survival and clinicopathological factors of ccRCC had statistically significant correlations [28]. We found a significant correlation between the tumor size and stage and the levels of FUBP1 mRNA expression in ccRCC tissues from patients. Our results indicated that patients with high FUBP1 expression developed larger ccRCC, which is consistent with the proliferative role for FUBP1 in ccRCC. However, our findings contradicted a study, which reported that the expression levels of FUBP1 protein and the clinicopathological characteristics were not correlated despite the highly significant association between high levels of FUBP1 protein and high tumor cell proliferation rates (ki-67) [35]. We speculate that different methods used to detect the levels of FUBP1 mRNA or proteins and different samples included may contribute to these discrepancies.

A recent study suggested that FUBP1 suppressed cell proliferation by negatively regulating NPM translation via its interaction with the NPM 3´-UTR [36]. In oligodendrogliomas FUBP1 expression was abolished because of mutations and it was considered a tumor suppressor [20, 37, 38]. However, these studies seemed contradictory to other studies where FUBP1 exerted a cancer-promoting function during tumorigenesis. Ding et al. reported that FUBP1 may promote growth in glioma cells by regulating the c-myc and the G1/S transition [30]. Singer et al. found that stathmin and stathmin-like 3 (SCLIP) could induce tumor cell proliferation, migration, and matrix invasion in NSCLC cell lines, FUBP1 might be as a pivotal inducer of stathmin and SCLIP [25]. Furthermore, the present study provided evidence that FUBP1 expression was upregulated in ccRCC. FUBP1 also promoted the growth of ccRCC cells in vitro. Therefore, FUBP1 plays an oncogenic role in ccRCC. These findings indicate that FUBP1 may have a dual functions in different cancers, and further studies on FUBP1 are required.

FUBP1 is a multifunctional protein depending on its subcellular localization [11]. It primarily abides within the nucleus as a transcription regulator, which is conferred by three nuclear localization signals (NLS), including a canonical bipartite NLS in the N-terminal and two atypical signals: an α-helix in the central domain and a tyrosine-rich motif in the C-terminal domain [39, 40]. FUBP1 recognizes and binds to the sequence of single-strand FUSE in target gene promoters and acts through the FUSE/FUBP1/FIR/TFIIH system to induce and fine-tune target gene expression. For example, FUBP1 was recruited to the FUSE sequence, which is more than 1500 base pairs upstream from the c-myc promoter P1 and potentiated c-myc expression [10, 13, 14, 16, 30, 31, 35, 40–44]. Rabenhorst et al. found that the human p21 promoter had four potential FUBP1 binding sites within 3.2 kb upstream of its transcription start site, and demonstrated that FUPB1 was bound to the p21 promoter and directly regulated its activity in HCC [24]. A better match with the optimal FUBP1-binding element, than found at the c-myc FUSE, was located at 2.5 kb upstream of the USP29 transcription start point, JTV1 acted via FUBP1 to upregulate USP29 transcription [45]. In addition to c-myc, p21, and USP29, two researches showed that the expression levels of stathmin mRNA were strongly correlated with that of FUBP1 mRNA in HCC and NSCLC [21, 25]. FUBP1 was also identified as a splicing regulator, which facilitated the efficient splicing of MDM2 pre-mRNA or induced exon exclusion [46, 47]. FUBP1 could directly and physically interact with p53 and suppress p53 transactivation activity under radiation treatment [29]. Under various stimuli, such as viral infection and apoptosis, FUBP1 could translocate from the nuclei to the cytoplasm, which contributes to mRNA stability and translation, such as GAP-43, p27, and NPM, as well as the replication of viruses, such as the Japanese encephalitis virus and hepatitis C virus [36, 48–52]. We observed that FUBP1 was almost exclusively expressed in the nucleus of cells and positively correlated with c-myc and p21 mRNA expression. c-myc is a potent proto-oncogene that is overexpressed and/or activated in more than half of human cancers, the gene is implicated in tumor initiation, progression, and maintenance [53]. As a global transcription factor, c-myc is thought to regulate 10%–15% of genes in human genome [54, 55]. These genes are involved in cell cycle progression, apoptotic cell death, glycolysis, glutaminolysis, and lipid synthesis. By employing fluorescence in situ hybridization, the deletion at 3p25.1–25.3 with c-myc gain had a significant correlation with tumor size in ccRCC [56]. Furthermore, an in vitro study indicated that c-myc knockdown significantly inhibited ccRCC cell proliferation by arresting the cell cycle in the G0/G1 phase [57]. Meanwhile, the use of a conditional transgenic mouse model revealed that c-myc could cause RCC by alternating glutamine metabolism [58]. p21, which is the founding member of the Cip/Kip family of CKIs, can bind and inhibit a range of cyclin/Cdk complexes, thereby leads to cell cycle arrest. Meanwhile, mounting evidence suggests that p21 is a major inhibitor of apoptosis [59]. p21 nuclear and cytosolic positivity correlated with the tumor grade in ccRCC, and cytosolic p21 expression in the primary tumor was an independent indicator of poor prognosis in patients who had metastatic disease [60]. However, an in vitro study revealed that inducing p21 expression decreased the viability of cancer cells via G1 arrest and apoptosis in A-498 cells [61]. So, it is still needed to study which genes are transcriptionally regulated by FUBP1 in ccRCC and how FUBP1 coordinates the activity of these genes.

In conclusion, our study has identified FUBP1 as a potential oncogene in ccRCC, which may contribute to the development of a novel biomarker or a promising anticancer therapy.
